# Undergraduates’ workplace learning in health sciences education: psychometric properties of single-item measures

**DOI:** 10.1186/s12909-024-05848-7

**Published:** 2024-08-10

**Authors:** Evelyn Steinberg, Takuya Yanagida, Stephan Marsch, Laura Dörrenbächer-Ulrich, Lukas Schwarz, Ulrike Auer, Christin Kleinsorgen, Christopher Pfeiffer, Petra Bührle, Franziska Perels

**Affiliations:** 1 Vice-Rectorate for Study Affairs and Clinical Veterinary Medicine, University of Veterinary Medicine Vienna, Veterinaerplatz 1, Vienna, 1210 Austria; 2 Department of Educational Science, Saarland University, Campus A4 2, 66123 Saarbrücken, Germany; 3 Clinic for Swine, University of Veterinary Medicine Vienna, Veterinaerplatz 1, Vienna, 1210 Austria; 4 Clinic for Small Animals, University of Veterinary Medicine Vienna, Veterinaerplatz 1, Vienna, 1210 Austria; 5 Centre for E-Learning, Didactics and Educational Research, University of Veterinary Medicine, Bünteweg 11, 30559 Hannover, Germany

**Keywords:** Single items, Workplace learning, Self-regulated learning, Cognition, Learning strategies, Motivation, Emotions, Learning environment, Learning context

## Abstract

**Background:**

Undergraduates’ workplace learning is an important part of health sciences education. Educational psychology research considers many different aspects of self-regulated learning at the workplace, including cognition, motivation, emotions, and context. Multivariate longitudinal and diary studies in this field require fewer items than alternatives or even a single item per construct and can reveal the sub-processes of workplace learning and contribute to a better understanding of students’ learning. Short instruments are necessary for application in workplace settings, especially stressful ones, to mitigate survey fatigue. The present study aimed to assess the psychometric properties of single items measuring various aspects of workplace learning.

**Methods:**

Twenty-nine single items selected from the Workplace Learning Inventory in Health Sciences Education were analyzed for reliability, information reproduction, and relationships within the nomological network. The authors additionally analyzed four generally formulated single items’ relationships with the full Workplace Learning Inventory scales and external criteria within the nomological network. Participants were 214 ninth- or tenth-semester veterinary medicine students in Austria and Germany who were learning at varied workplaces during the winter semester of 2021/2022.

**Results:**

Of the 29 single items selected from existing scales, 27 showed sufficient reliability, but mixed results were obtained regarding validity. Although the items’ relationships within the nomological network were similar to those of the full scales, information reproduction was insufficient for most items. The four general single items showed acceptable validity, but the reliability of these measures of states could not be assessed.

**Conclusions:**

This paper reported findings on the psychometric properties of single items for undergraduates’ workplace learning in health science education. The findings are crucial for deciding whether to use scales versus single-item measures in future studies. By applying the findings, researchers can be more economical in their workplace learning data collection and can include more constructs.

**Supplementary Information:**

The online version contains supplementary material available at 10.1186/s12909-024-05848-7.

## Introduction

Health sciences education has long focused on the science of teaching, but in recent years we have seen a shift toward the science of learning [1, 2]. This study focused on undergraduates’ workplace learning in health sciences education [3–5] from an educational psychology perspective. Psychological theories, especially the theory of self-regulated learning (SRL), focus on the individual learner and view learning as a process in which cognitive, motivational, emotional, and contextual aspects are considered [6–8]. To gain a better understanding of undergraduates’ learning processes at the workplace, multivariate and longitudinal studies are needed. Such studies require fewer items per construct, which helps avoid survey fatigue and increases applicability in workplace settings, especially stressful ones.

We assessed the psychometric properties of single-item measures of constructs related to self-regulated learning at the workplace and this paper discusses the items’ role in health sciences education research. We selected some single items from the Workplace Learning Inventory in Health Sciences Education (WLI) [9] scales and specifically developed others using more general wording.

### Self-regulated learning in the workplace

**Fig. 1:**
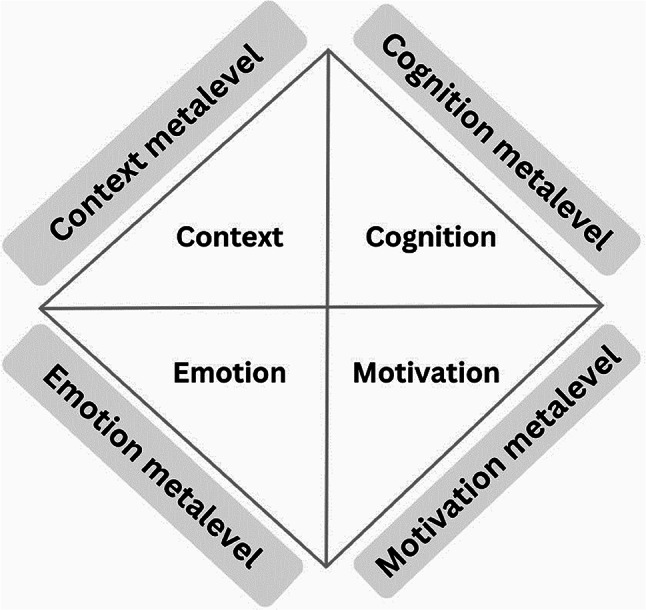
The four areas of workplace SRL at the learning process level (inner components) and the metalevel (outer components), based on Steinberg et al. [9]

The study was based on SRL research [10, 11]. In health sciences education, the most prominent SRL theory is a process-based model, namely Zimmerman’s cyclical phases model, which differentiates between the forethought, performance, and reflection phases [12, 13]. Besides process-based models, there are also component-based models that integrate different areas (e.g., Pintrich’s conceptual framework for assessing motivation and SRL) [14] and levels (e.g., Boekaerts’ six-component model of SRL) [15]. The present study adopted Steinberg et al.‘s [9] component-based model as its conceptual framework. The model integrates four areas, namely cognition, motivation, emotion, and context, at two levels, namely the learning process level and the metalevel, resulting in a total of eight components (see Fig. 1).

Cognition refers to learning strategies focused on workplace learning [16]. Motivation means instigating and sustaining goal-directed activity [17]. Emotions are defined within the broader concept of affect but are distinguished from other affective phenomena, such as moods, in that emotions are more intense, have a clearer object focus and a more salient cause, and are typically experienced for a shorter duration [18–20]. Context means undergraduate medical students’ perceptions of multiple dimensions of the educational environment in the clinical practice setting [21]. The metalevel of cognition, motivation, emotion, and context means regulating those respective aspects of the learning process [14, 22–25]. For more details on the model, we refer to Steinberg et al. [9].

Steinberg et al. identified aspects relevant to the eight components of undergraduates’ workplace learning and developed corresponding scales, resulting in the WLI [9], which provides 31 scales, each comprising three to six items. Researchers investigating workplace learning can select the scales that are relevant to their research questions. Table 1 lists the constructs with their corresponding definitions.

Van Houten-Schat et al. [13] and Roth et al. [26] reviewed SRL research and respectively identified the need to investigate SRL sub-processes in the workplace to gain a better understanding of the interplay of the different SRL aspects, as well as the need to use more diverse methodologies in SRL research, including multivariate longitudinal and diary studies. Single-item measures of the WLI constructs could facilitate such studies.

**Table 1:** WLI constructs and definitions (based on Steinberg et al.) [9]

Construct	Definition
Cognition	
Cognitive learning strategies	…refer to the learning and practice of professional medical activities.
Preparation	…means activating knowledge as well as subject-related preparation regarding professional medical activities before entering the clinical practice setting.
Attention	…means focusing on and learning from observing or performing professional medical activities in the clinical practice setting.
Rehearsal	…means repeating and memorizing important facts and/or mentally replaying important procedures in the clinical practice setting.
Elaboration	…means integrating new information into one’s existing information structure in the clinical practice setting.
Clarification	…means clarifying unclear aspects or asking for support regarding professional medical activities that can be directly applied in the short run during learning in the clinical practice setting.
Consolidation	…means processing experiences and new knowledge regarding professional medical activities after learning in the clinical practice setting.
Proximal metacognitive learning strategies	…are strategies through which students learn from regulating professional medical activities.
Planning	…means anticipating and planning professional medical activities before entering the clinical practice setting.
Reviewing	… means briefly pausing in the clinical practice setting to assess the clarity of a professional medical activity (and related theoretical foundations and practical processes).
Reflection	…means reflecting on experiences of clinical practical activities after learning in the clinical practice setting.
Motivation	
Expectancy of success	…means the individuals’ beliefs about how well they will do in an upcoming professional medical activity.
Situational interest	…means liking and willfully engaging in practicing and learning
Mastery goal approach	…means focusing on attaining task-based or intrapersonal competence.
Performance goal approach	…means focusing on attaining normative competence.
Effort	…means persevering with practicing and learning even when it is difficult.
Attention control	…means not getting distracted from practicing and learning.
Proactive attitude	…means seeking and taking opportunities to practice and learn.
Emotion	
Negative emotions	…include fear/anxiety, frustration, anger, and sadness.
Positive emotions	…include pride, happiness, curiosity, and hope.
Context	
Organizational framework conditions	…are students’ perceptions of the workplace and staff’s preparedness for their integration.
Supervisory quality	…means students’ perceptions of the learning environment shaped by the supervisor.
Staff support	…means students’ perceptions of the learning environment shaped by the staff.
Peer support	…means students’ perceptions of the learning environment shaped by their peers.
Equal treatment	…means students’ perceptions of the diversity culture.
Cognition metalevel	
Monitoring	…means monitoring the expediency of the implemented (meta-)cognitive learning strategies.
Control	…means changing the cognitive learning strategies when problems are encountered.
Motivation metalevel	
Monitoring	…means monitoring the expediency of the motivation level and direction.
Control	…means changing the motivation level and direction when problems are encountered.
Emotion metalevel	
Monitoring	…means monitoring the expediency of the emotion quality and intensity.
Control	…means changing the emotion quality and intensity when problems are encountered.
Context metalevel	
Monitoring	…means monitoring whether the learning environment is perceived as supportive.
Control	…means adapting to a difficult learning environment or changing the contextual aspects when problems are encountered.

The table is based on Steinberg et al. [9]

### Single-item measures

We summarize the discussion on the advantages and disadvantages of using single items in scientific studies, based on overviews provided in the literature [27–29]. Arguments in favor of the use of single items include parsimony, which is relevant in holistic studies considering the large number of theoretical constructs, as well as in diary studies with many measurement points and in time-limited settings such as data collection in the workplace. Parsimony is also associated with increased participant motivation and cognitive involvement, resulting in fewer missing values and higher validity. Moreover, parsimony addresses researchers’ ethical commitment to participants; that is, researchers strive not to overburden participants and to avoid their confusion and frustration when answering similar items. Other arguments in favor of the use of single items are their lower ambiguity, better interpretability, higher face validity, and reduced risk of criterion contamination.

Arguments against the use of single items include their lower or unknown reliability, their inability to adequately capture complex psychological constructs, and the less fine-grained distinctions between individuals. Hence, single items are usually acceptable when the construct is concrete, unidimensional, clearly defined, narrow in scope, and used as a moderator or control variable or when the desired precision is low [27, 28, 30]. Fisher et al. summarized successful examples of single items used in organizational psychology [28].

If there is uncertainty about whether a construct meets the above requirements, validation tests can be performed to ensure trustworthiness [30]. The appropriate validation method depends on how the item was developed, that is, whether it was selected from an existing scale or developed anew [28]. For items selected from a scale, Gogol et al. [29] have provided the following best practice for examining the psychometric properties: [27] assessing the reliability, information reproduction, and relationships within the nomological network. For newly developed single items measuring stable characteristics (or traits), the recommendation is to assess the test–retest reliability [28]; however, this method is inappropriate for single items measuring states that are expected to change over time, as in longitudinal studies [31]. To provide evidence of the validity of newly developed single items, assessing relationships within the nomological network is recommended.

### Aim

Our study aimed to examine two sets of single items appropriate for research on undergraduate health science students’ learning by analyzing their reliability, their correspondence to the full scale, and their relations with external criteria. These sets of single items could be helpful for economically conducting multivariate longitudinal and intensive longitudinal studies in health workplace settings. First, we investigated 29 single items selected from the WLI [9]. The items address four areas of workplace learning, namely cognition, motivation, emotion, and context, at two levels, namely the learning process level and the metalevel. Each of the eight components is represented by several items, with the exception of emotion on the learning process level since Duffy et al. [20] have already provided single items for that. We systematically compared the single items with their corresponding full scales with respect to the following measurement questions [29]: (1) How reliable are single-item measures? (2) How well do single-item measures reproduce the information that the full scales obtain? (3) How well do single-item measures reproduce the relationships with external criteria in the nomological network that the full scales obtain?

Second, we examined four newly developed and more generally formulated single items measuring states rather than traits [32]. The items represent cognition, motivation, emotion, and context at the learning process level. Although their reliability cannot be tested, we examined the items’ validity with respect to the following measurement questions: (1) How well do single-item measures correlate with their respective full WLI scales? (2) How well do single-item measures relate to external criteria within the nomological network?

## Methods

### Participants

The outcomes should represent a diverse population of undergraduate health sciences students in the aspects of cognition, motivation, emotion, and learning environment. Consequently, we made a deliberate effort to encompass the majority of a pertinent student cohort from a single institution rather than distributing a questionnaire to students at different institutions, which could have led to a biased sample predominantly comprising highly motivated high achievers. We invited students from a second institution to participate in achieving the predetermined target sample size of *n* = 200 in adherence to a rule of thumb guideline for the minimum sample size for confirmatory factor analysis [33].

Participants were from two higher education institutions in Austria and Germany. At Institution 1, the target group comprised 200 students enrolled in a Clinical Rotation course as part of a veterinary degree program in which students learn in a clinical practical setting over a relatively long period for the first time. Students take this course in their ninth semester and rotate among highly varied workplace settings (rotations include, e.g., anesthesia/imaging diagnostics, surgery, gynecology, internal medicine, emergency department, reproduction). Although all 200 enrollees participated in the study, 13 students did not consent to their data being used for research purposes, and 11 consenting participants had to be excluded from further analysis owing to a high proportion of missing values (> 50%); the final sample size was *n* = 176 at Institution 1.

At Institution 2, the target group comprised about 260 students in their practical year of a veterinary degree program. Students usually complete their practical year during the ninth and tenth semesters and familiarize themselves with various workplaces. The questionnaire was opened 91 times; thereof 38 participants completed more than 50% of the items and consented to their data being used for research purposes. Combining both samples, the total sample size was *n* = 214 (78% [167] female[s], 21% [45] male[s], 1% [2] diverse; age: 21–41 years; M = 24.79, SD = 2.74). There were no statistically significant differences in gender (female: 77.90% [167] and 79.37% [170]) and age (M = 24.76, SD = 2.67 and M = 24.56, SD = 3.99) between Institutions 1 and 2, respectively.

### Measures

We tested the psychometric properties of two sets of single items. First, the project team selected 29 single items from the WLI’s 29 full scales [9]. The project team comprised one professor, three senior scientists, two clinical teachers, and two students, all working in health science education and/or educational psychology. Single items were selected based on content and factor loadings (using the data of the study at hand). We preferred items couched in broader terms and considered face validity according to the project team’s ratings as well as those of nine researchers in the field of health sciences education and/or SRL who were not part of the project team. Furthermore, we chose items with high factor loadings (see Steinberg et al. [9] for details on factor loadings). Second, we tested the psychometric properties for four generally formulated single items representing cognition, motivation, emotion, and context on the learning process level. The project team developed these items using established instruments/scales and experiences with the SRL questionnaire and diary items, as well as theoretical assumptions. Table 2 provides an overview of the items.

**Table 2:** Overview of single-items

Name of single item	Single-item – English (back- and forth translation)	Single-item – German (original)	Item label
Cognition general	I am learning and practicing today in a way that will allow me to take away as much as possible.*	Ich lerne und übe heute in einer Art und Weise, dass ich möglichst viel mitnehmen kann.*	
Preparation	Before I came to the workplace, I worked to acquaint myself with relevant topics.	Bevor ich in die Klinik bzw. in den Betrieb kam, habe ich mich in relevante Themen eingearbeitet.	Pre1
Attention	At the workplace, I stayed concentrated while conducting practical medical tasks.	Vor Ort in der Klinik bzw. im Betrieb war ich bei der Durchführung von medizinisch-praktischen Tätigkeiten konzentriert.	Att1
Rehearsal	At the workplace, I consciously committed important information to memory.	Vor Ort in der Klinik bzw. im Betrieb habe ich mir Wichtiges bewusst eingeprägt.	Reh1
Elaboration	At the workplace, I tried to connect the practical medical tasks to what I had previously learned.	Vor Ort in der Klinik bzw. im Betrieb habe ich versucht, die medizinisch-praktischen Tätigkeiten mit dem, was ich bisher gelernt habe, zu verbinden.	Ela1
Clarification	At the workplace, I asked for advice when something was unclear.	Vor Ort in der Klinik bzw. im Betrieb habe ich bei Unklarheiten um Rat gefragt.	Cla1
Consolidation	After leaving the workplace (no matter if e.g., 10 min or 2 h afterwards), I further deepened what I had learned and practiced.	Nach Verlassen der Klinik bzw. des Betriebes (egal ob z.B. 10 min oder 2 h danach), habe ich das, was ich gelernt und geübt habe, nochmals vertieft.	Con1
Planning	Before I came to the workplace, I thought about what medical cases I could expect.	Bevor ich in die Klinik bzw. in den Betrieb kam, habe ich überlegt, welche medizinischen Fälle mich erwarten.	Pla1
Reviewing	At the workplace, I recapitulated what I had practiced or learned in order to determine whether everything is clear to me.	Vor Ort in der Klinik bzw. im Betrieb, habe ich das Geübte oder Gelernte rekapituliert, um festzustellen, ob mir alles klar ist.	Rev1
Reflection	After leaving the workplace (no matter if e.g., 10 min or 2 h afterwards), I reflected on what I would do differently next time.	Nach Verlassen der Klinik bzw. des Betriebes (egal ob z.B. 10 min oder 2 h danach), habe ich nachgedacht, was ich nächstes Mal anders machen würde.	Ref1
Motivation general	I am motivated today.*	Ich bin heute motiviert.*	
Expectancy of success	I am confident that this week I will be able to do what is asked of me.	Ich bin zuversichtlich, dass ich diese Woche das, was gefordert wird, umsetzen kann.	EoS1
Situational interest	This week I found the tasks interesting.	Diese Woche habe ich die Aufgaben interessant gefunden.	SiI1
Mastery goal approach	This week it was important to me to expand my knowledge.	Diese Woche war es mir wichtig, mein Wissen zu erweitern.	MaA1
Performance goal approach	This week it was important to me to practice exactly what the instructors are looking for when evaluating my performance.	Diese Woche war es mir wichtig, genau das zu üben, worauf es Lehrenden bei der Beurteilung meiner Leistung ankommt.	PeA3
Effort	This week I made an effort.	Diese Woche habe ich mich angestrengt.	Eff1
Attention control	This week I was not concentrated while practicing and studying.+	Diese Woche war ich beim Üben und Lernen unkonzentriert.+	AtC1
Proactive attitude	This week I took advantage of opportunities to gain hands-on practice.	Diese Woche habe ich Möglichkeiten zum praktischen Üben genutzt.	PrA1
Emotion general	I am feeling good while learning and practicing today.*	Ich fühle mich heute gut beim Lernen und Üben.*	
Context general	I have good contextual conditions^a^ for studying and practicing in the clinic / workplace today. ^a^(organisational conditions, instructors, other students, on-site staff, equity concerns) *	Ich habe heute in der Klinik bzw. im Betrieb gute Rahmenbedingungen^a^ für das Lernen und Üben. ^a^(Organisatorische Rahmenbedingungen, Lehrende, Mitstudierende, Team vor Ort, Gleichbehandlung)*	
Organizational framework conditions	I had the impression that the clinic / facility was well-organized, so that students encountered good contextual conditions.	Ich hatte den Eindruck, dass die Klinik bzw. der Betrieb gut organisiert war, so dass Studierende gute Rahmenbedingungen vorfanden.	Ofc1
Supervisory quality	The instructors offered me opportunities to further develop.	Die Lehrenden boten mir Gelegenheiten, mich weiterzuentwickeln.	SuQ1
Staff support	I was supported by members of the staff working here.	Ich hatte Unterstützung von Personen aus dem Team, das hier arbeitet.	StS1
Peer support	I had the impression that the students support each other.	Ich hatte den Eindruck, dass sich die Studierenden gegenseitig unterstützen.	PeS1
Equal treatment	All students were treated equally regardless of gender.	Alle Studierenden wurden unabhängig von ihrem Geschlecht gleich behandelt.	EqT1
Monitoring cognition	This week I paid attention to whether my studying and practicing behavior would help me reach my goal.	Diese Woche habe ich darauf geachtet, ob mein Lern- und Übungsverhalten zielführend ist.	CoM1
Control cognition	This week I changed the way I study or practice when I noticed that I was not getting better.	Diese Woche habe ich die Art und Weise, wie ich lerne oder übe, geändert, wenn ich bemerkt habe, dass ich nicht besser werde.	CoC1
Monitoring motivation	This week I paid attention to how motivated I am.	Diese Woche habe ich darauf geachtet, wie motiviert ich bin.	MoM1
Control motivation	This week I changed something when I noticed that I was not motivated.	Diese Woche habe ich etwas geändert, wenn ich gemerkt habe, dass ich nicht motiviert bin.	MoC1
Monitoring emotion	This week I reflected on my feelings while studying and practicing.	Diese Woche habe ich über meine Gefühle beim Lernen und Üben nachgedacht.	EmM1
Control emotion	This week I changed something when I noticed that my feelings (e.g., fear or anger) were impeding me while studying or practicing.	Diese Woche habe ich etwas geändert, wenn ich gemerkt habe, dass mich meine Gefühle (z.B. Angst oder Ärger) beim Lernen oder Üben beeinträchtigen.	EmC1
Monitoring context	This week I reflected on what contextual conditions^a^ accompany my studying and practicing. ^a^(organisational conditions, instructors, other students, on-site staff, equity concerns)	Diese Woche habe ich darüber nachgedacht, welche Rahmenbedingungen^a^ mein Lernen und Üben begleiten. ^a^(organisatorische Rahmenbedingungen, Lehrende, Mitstudierende, Team vor Ort, Gleichbehandlung)	CnM1
Control context	This week I changed how I study or practice in order to better adapt to contextual conditions^a^. ^a^(organisational conditions, instructors, other students, on-site staff, equity concerns)	Diese Woche habe ich die Art und Weise, wie ich lerne oder übe, geändert, um mich an die Rahmenbedingungen^a^ besser anzupassen. ^a^(organisatorische Rahmenbedingungen, Lehrende, Mitstudierende, Team vor Ort, Gleichbehandlung)	CnC1

All items were administered using a unipolar 5-point Likert-type response format where 1 = D*oes not apply at all*, 2 = *Does not apply*, 3 = P*artly applies*, 4 = *Applies*, and 5 = *Fully applies*, for the control scales at the metalevel, 6 = *This case did not occur* was also included. *Set 2: Generally formulated items. +Reverse coded. The English translation is based on back- and forth translation performed by two different translators, as is recommended for translating questionnaires. The table is based on Steinberg et al., to whose work the item labels also refer [9]

To validate the single items, we used the WLI, as well as measures of external criteria within the nomological network, which have also been used to validate the WLI [34–42]. A nomological network is a system of related constructs [43]. We excluded external criteria for emotions at the learning process level and context at the metalevel because none were available in German. Table 3 provides an overview of the measures.

**Table 3:** Overview of measures

Component	Set 1 – single-item measures selected from full WLI scales	Set 2 – generally formulated single-item measures (newly developed)	Full WLI scales (number of items)	Scales measuring external criteria (number of items)
Cognition				
Cognitive learning strategies	Preparation Attention Rehearsal Elaboration Clarification Consolidation	Cognition general	Preparation (4) Attention (5) Rehearsal (5) Elaboration (5) Clarification (5) Consolidation (5)	Organization (3) Elaboration (3) Critical review (3) Rehearsal (3) Literature research (3) [34]
Proximal metacognitive learning strategies	Planning Reviewing Reflection		Planning (5) Reviewing (5) Reflection (5)	Goalsetting/Planning (6) Control (6) Regulation (8) [35]
Motivation	Expectancy of success Situational interest Mastery goal approach Performance goal approach Effort Attention control Proactive attitude	Motivation general	Expectancy of success (5) Situational interest (5) Mastery goal approach (5) Performance goal approach (5) Effort (3) Attention control (3) Proactive attitude (5)	Self-efficacy (4) [35] Learning goal approach (4) Performance goal approach (4) Performance goal avoidance (4) [36] Attention control (3) [35]
Emotion		Emotion general	Negative emotions (4) Positive emotions (4)	
Context	Organizational framework conditions Supervisory quality Staff support Peer support Equal treatment	Context general	Organizational framework conditions (5) Supervisory quality (6) Staff support (5) Peer support (5) Equal treatment (4)	Perception of teachers (11) Perception of atmosphere (12) [38, 39]
Cognition metalevel	Monitoring Control		Monitoring (5) Control (5)	Goalsetting/Planning (6) Control (6) Regulation (8) [35]
Motivation metalevel	Monitoring Control		Monitoring (5) Control (5)	Increasing situational interest (5) Increasing personal value (3) Performance-goal-approach oriented self-instruction (5) Self-rewarding (4) Mastery-goal-approach oriented self-instruction (4) Controlling learning environment (3) Performance-goal-avoidance oriented self-instruction (3) Setting subgoals (3) [40]
Emotion metalevel	Monitoring Control		Monitoring (5) Control (5)	Self-incrimination (Self-blame) (3) Acceptance (3) Rumination (3) Positive refocusing (3) Refocus(ing) on planning (3) Positive reevaluation (reappraisal) (3) Relativize (putting into perspective) (3) Catastrophize(3) Accusing (blaming) others (3) [41, 42]
Context metalevel	Monitoring Control		Monitoring (5) Control (5)	

^a^Items and response formats for external criteria were slightly adapted from the original questionnaires, where necessary (e.g., “At school” was replaced by “In my studies”)

^b^To avoid overextending the students, all scales were administered using the same 5-point Likert scale, where 1 = *Does not apply at all*, 2 = *Does not apply*, 3 = *Partly applies*, 4 = *Applies*, and 5 = *Fully applies*, except for the emotion component, for which 1 = *Not at all*, 2 = *A little*, 3 = *Moderately*, 4 = *Fairly*, and 5 = *Very much*

#### Procedure

At Institution 1, the students completed the questionnaires as part of the course, as it was an exercise that supported the course’s learning goal of “reflecting on one’s own learning and practice.” Due to the large number of items, we spread data collection over a week, and students completed the questionnaires in the period December 6–10, 2021, or December 13–17, 2021, using the online survey tool unipark© [44]. Each item was answered once. Every morning during the survey period, the participants received an email with an invitation link to the questionnaire and were encouraged to complete it in the workplace.

At Institution 2, the rectorate invited all students in their practical year to participate in the study via an email with a link to the online questionnaire. Students were allowed to pause at any time and continue completing the questionnaire later within the aforementioned period using the online survey tool unipark© [44]. To improve the response rate at Institution 2, participants who completed the questionnaire were entered into a raffle to win a €50 voucher.

To avoid survey fatigue, the following steps were taken in addition to spreading the data collection over a week: Students were provided with targeted information about the study’s aims and benefits; teachers gave students time to complete the survey at their workplace; and students received individual feedback on their results, with tips and tricks for further developing their SRL skills.

### Data analysis

We analyzed the first set of single items, which were selected from existing scales, according to Gogol et al.’s [29] recommendations for single-item measures in psychological science [27]. Accordingly, we assessed (1) the items’ reliability by computing the coefficient ω reflecting the proportion of item variance accounted for by the latent construct (Note that the items‘ reliability is the square of the standardized factor loading, see Brown (, p.115) [45], (2) the amount of reproduced information by computing the product–moment correlation between the scores obtained by the full scales and the scores for every single item, while accounting for the overlapping error variance, [46] and (3) relationships within the nomological network by computing product–moment correlations between the single-item measures and measures of external criteria within the nomological network. Similarly, we examined the second set of single items, which were generally formulated, by assessing their relationships with the full WLI scales and their relationships within the nomological network. These items’ reliability could not be assessed because they were not derived from a scale; their ω could not be calculated, and test–retest reliability is inappropriate for measures of states. Note that there is no clear cut-off separating good and poor reliability, but it has been suggested that 0.70 is an acceptable lower bound [47], with values between 0.65 and 0.70 considered minimally acceptable [48]. For correlations, *r* = .10 is considered as small, *r* = .30 as medium and *r* = .50 as large [49]. Analyses were conducted in Mplus 8.6 (Muthén and Muthén, Los Angeles, California) and R 4.3.1 (R Core Team, Vienna, Austria) [50, 51]. All analyses are based on the significance level $\:\alpha\:$ = 0.05.

## Results

Table 4 provides an overview of the detailed results, after which we summarized the results for the 29 single items selected from the full WLI [9] scales, as well as those for the four generally formulated single items.

**Table 4:** Psychometric characteristic of the full-scale (FS) and single-item (SI), including reliability, correlation with full scale (information reproduction) and correlation with external criteria (nomological network)

Cognition																	
Cognitive learning strategies	Preparation		Attention		Rehearsal		Elaboration		Clarification			Consolidation			Cognition general		
	FS	SI	FS	SI	FS	SI	FS	SI	FS	SI	FS		SI		SI		
Reliability, $\:\omega\:$	0.867	0.745	0.891	0.731	0.787	0.694	0.753	0.828	0.713	0.841	0.859		0.666				
Correlation with full scale, *r*		**0.580**		**0.534**		**0.413**		**0.623**		**0.481**			**0.476**				
Correlation with cognition general (SI), *r*	**0.377**		**0.403**		**0.485**		**0.510**		**0.426**		**0.390**						
Correlation with external criterion																	
Organization, *r*	**0.163**	0.101	0.111	0.062	0.145	0.120	**0.179**	0.152	0.073	− 0.053	**0.212**		0.131		0.101		
Elaboration, *r*	**0.167**	0.088	**0.288**	**0.281**	**0.266**	**0.287**	**0.357**	**0.377**	**0.318**	**0.198**	**0.210**		**0.232**		**0.215**		
Critical review, *r*	**0.239**	**0.191**	0.105	0.103	**0.208**	**0.243**	**0.264**	**0.248**	**0.244**	0.100	**0.252**		**0.290**		0.175		
Rehearsal, *r*	0.087	0.109	− 0.059	− 0.075	0.054	− 0.076	− 0.121	− 0.121	**− 0.167**	− 0.197	0.068		0.043		− 0.086		
Literature research, *r*	**0.320**	**0.268**	**0.208**	0.126	**0.263**	**0.268**	**0.209**	**0.217**	**0.361**	**0.239**	**0.231**		0.150		0.118		
Proximal metacognitive learning strategies	Planning		Reviewing		Reflection		Cognition general										
	FS	SI	FS	SI	FS	SI	SI										
Reliability, $\:\omega\:$	0.695	0.706	0.693	0.588	0.818	0.755											
Correlation with full scale, *r*		**0.358**		**0.199**		**0.552**											
Correlation with cognition general (SI), *r*	**0.157**		**0.415**		**0.279**												
Correlation with external criterion																	
Goal setting and planning, *r*	0.145	0.076	0.096	0.125	0.079	0.013	− 0.008										
Control, *r*	0.075	0.089	0.165	.**249**	0.151	0.139	**0.197**										
Regulation, *r*	0.065	0.040	**0.183**	0.144	0.047	0.042	0.067										
Motivation	Expectancy of success		Situational interest		Mastery goal approach		Performance goal approach		Effort			Attention control			Proactive attitude		Motivation general
	FS	SI	FS	SI	FS	SI	FS	SI	FS	SI	FS		SI		FS	SI	SI
Reliability, $\:\omega\:$	0.895	0.745	0.883	0.769	0.923	0.771	0.828	0.778	0.659	0.757	0.842		0.740		0.804	0.832	
Correlation with full scale, *r*		**0.580**		**0.632**		**0.627**		**0.529**		**0.514**			**0.589**			**0.671**	
Correlation with motivation general (SI), *r*	**0.196**		**0.672**		**0.638**		**0.181**		**0.518**		**− 0.509**				**0.439**		
Correlation with external criterion																	
Self-efficacy, *r*	**0.463**	**0.336**	0.054	0.065	0.130	0.091	− 0.115	− 0.127	0.036	− 0.005	− 0.207		− 0.242		0.131	0.080	0.044
Learning goal approach, *r*	**0.376**	**0.348**	**0.269**	**0.242**	**0.401**	.**376**	0.096	0.025	**0.379**	**0.326**	− 0.113		− 0.137		**0.360**	**0.321**	**0.316**
Performance goal approach, *r*	0.051	0.114	0.070	0.024	0.067	0.037	**0.242**	0.129	− 0.041	− 0.059	− 0.003		− 0.024		0.058	0.059	0.051
Performance goal avoidance, *r*	− 0.016	0.060	0.094	0.021	0.066	− 0.001	**0.271**	0.110	0.011	0.004	0.061		0.019		− 0.008	0.036	0.022
Attention control, *r*	− 0.126	− 0.170	− 0.071	− 0.055	− 0.142	− 0.081	0.035	0.107	− 0.086	− 0.063	**0.312**		**0.300**		− 0.059	− 0.084	− 0.060
Emotion	Negative emotions		Positive emotions														
	FS		FS														
Reliability, $\:\omega\:$	0.792		0.788														
Correlation with emotion general (SI), *r*	**− 0.542**		**0.658**														
Context	Organizational framework conditions		Supervisory quality		Staff support		Peer support		Equal treatment			Context general					
	FS	SI	FS	SI	FS	SI	FS	SI	FS	SI	(SI)						
Reliability, $\:\omega\:$	0.874	0.814	0.849	0.694	0.859	0.724	0.855	0.679	0.761	0.783							
Correlation with full scale, *r*		**0.673**		**0.419**		**0.539**		**0.479**		**0.575**							
Correlation with context general (SI), *r*	**0.745**		**0.661**		**0.587**		**0.477**		**0.214**								
Correlation with external criterion																	
Perception of teachers, *r*	**0.707**	**0.617**	**0.684**	**0.568**	**0.715**	**0.621**	**0.537**	**0.467**	**0.465**	**0.358**	**0.609**						
Perception of atmosphere, *r*	**0.742**	**0.658**	**0.701**	**0.586**	**0.720**	**0.552**	**0.511**	**0.441**	**0.282**	**0.250**	**0.677**						
Cognition metalevel	Monitoring		Control														
	FS	SI	FS	SI													
Reliability, $\:\omega\:$	0.914	0.757	0.837	0.638													
Correlation with full scale, *r*		**0.606**		**0.403**													
Correlation with external criterion																	
Goalsetting/planning, *r*	**0.183**	**0.222**	0.121	0.221													
Control, *r*	0.146	0.175	0.138	0.182													
Regulation, *r*	**0.284**	**0.316**	.**208**	0.117													
Motivation metalevel	Monitoring		Control														
	FS	SI	FS	SI													
Reliability, $\:{\omega\:}^{2}$	0.881	0.724	0.752	0.659													
Correlation with full scale, *r*		**0.569**		**0.276**													
Correlation with external criterion																	
Increasing situational interest, *r*	**0.199**	0.124	**0.187**	0.131													
Increasing personal value, *r*	0.148	0.059	0.149	0.156													
Performance-goal-approach oriented self-instruction, *r*	0.162	0.041	0.082	0.100													
Self-rewarding, *r*	0.134	0.075	**0.164**	0.135													
Mastery-goal-approach oriented self-instruction, *r*	**0.285**	**0.219**	**0.321**	0.178													
Controlling learning environment, *r*	**0.271**	**0.222**	**0.264**	− 0.012													
Performance-goal-avoidance oriented self-instruction, *r*	**0.194**	0.108	− 0.021	0.003													
Setting subgoals, *r*	0.075	0.059	0.149	− 0.021													
Emotion metalevel	Monitoring		Control														
	FS	SI	FS	SI													
Reliability, $\:\omega\:$	0.929	0.876	0.687	0.687													
Correlation with full scale, *r*		**0.786**		**0.494**													
Correlation with external criterion																	
Self-incrimination (Self-blame), *r*	0.139	0.107	− 0.070	− 0.060													
Acceptance, *r*	0.161	0.112	0.190	**0.210**													
Rumination, *r*	**0.414**	**0.368**	0.169	**0.303**													
Positive refocusing, *r*	0.121	0.093	0.074	0.169													
Refocus(ing) on planning, *r*	**0.266**	**0.219**	**0.197**	**0.321**													
Positive reevaluation (reappraisal), *r*	0.157	0.106	**0.273**	**0.249**													
Relativize (putting into perspective), *r*	0.028	0.041	**0.183**	0.002													
Catastrophize, *r*	**0.182**	**0.145**	− 0.063	0.029													
Accusing (blaming) others, *r*	0.013	− 0.036	− 0.069	0.091													
Context metalevel	Monitoring		Control														
	FS	SI	FS	SI													
Reliability, $\:\omega\:$	0.924	0.834	0.868	0.658													
Correlation with full scale, *r*		**0.725**		**0.786**													

Abbreviations: FS = Full scale; SI = single item

^a^Statistically significant correlations at α = 0.05 are in bold

### Single items selected from full scales

The following paragraphs summarize the results for the 29 single items selected from full scales. Twenty items showed acceptable reliability (ω > 0.70), seven showed minimally acceptable reliability (ω = 0.65 to ω = 0.70), and two showed unacceptable reliability (ω < 0.65) [48]. Of the 29 reliability values for the single items, eight differed significantly from the corresponding full-scale value but showed minimally acceptable to relatively high values (ranging from ω = 0.67 to ω = 0.83).

Regarding information reproduction, the single items showed low to substantial correlations (corrected for shared error variance) [34] with the corresponding full scales, with r ranging from 0.20 for reviewing to 0.79 for monitoring on the emotion metalevel. Of the 29 correlations, 27 values were below 0.70, indicating insufficient information reproduction (we considered less than 50% information reproduction to be insufficient; information reproduction expressed as a percentage is the square of the correlation values); two values were above 0.70, indicating substantial information reproduction.

Regarding the relationships of the selected single items within the nomological network, the items showed patterns that were similar to those of the full scales in terms of their correlations with the external criteria, but the correlations were significant less often. A similar pattern was reflected in the small mean absolute differences between the correlations obtained for the full scales and single items (between − 0.03 for all cognition metalevel aspects and 0.09 for all contextual aspects). The respective differences in the correlations ranged from − 0.25 to 0.28, but only 7 of the 124 correlations between the single items and the external criteria differed significantly from the correlations between the corresponding full scales and these external variables. See Table 5 for an overview of the results.

### Generally formulated single items

The following paragraphs summarize the results for the four general single items shown in Table 4 (cognition general, motivation general, emotion general and context general). The correlations between the generally formulated single items (see lines “correlation with … general”) and their respective full scales (see columns “FS”) ranged between 0.16 and 0.51 for general cognition, − 0.51 and 0.67 for general motivation, − 0.54 and 0.66 for general emotion and 0.21 and 0.74 for general context.

Regarding the general single items’ (see column “… general”) relationships within the nomological network (see lines below “correlation with external criteria”), the items showed low to substantial correlations with external criteria. Analyses showed significant correlations between the general cognition single item and the external criterion elaboration (*r* = .22), single-item general motivation and the external criterion learning goal approach, and single-item general context and the external criteria perception of teachers (*r* = .61) and perception of atmosphere (*r* = .68). See Table 5 for an overview of the results.

**Table 5:** Overview of results

	Reliability^a^	Recommendation for interpretation of results based on information reproduction^b^	Relationship within the nomological network
Name of the single item			
Cognition (general)	-	-	Acceptable
Preparation	Acceptable	Single item wording	Acceptable
Attention	Acceptable	Single item wording	Acceptable
Rehearsal	Minimally acceptable	Single item wording	Acceptable
Elaboration	Acceptable	Single item wording	Acceptable
Clarification	Acceptable	Single item wording	Acceptable
Consolidation	Minimally acceptable	Single item wording	Acceptable
Planning	Acceptable	Single item wording	Acceptable
Reviewing	Not acceptable	Single item wording	Acceptable
Reflection	Acceptable	Single item wording	Acceptable
Motivation (general)	-	-	Acceptable
Expectancy of success	Acceptable	Single item wording	Acceptable
Situational interest	Acceptable	Single item wording	Acceptable
Mastery goal approach	Acceptable	Single item wording	Acceptable
Performance goal approach	Acceptable	Single item wording	Acceptable
Effort	Acceptable	Single item wording	Acceptable
Attention control	Acceptable	Single item wording	Acceptable
Proactive attitude	Acceptable	Single item wording	Acceptable
Emotion (general)	-	-	Acceptable
Context (general)	-	-	Acceptable
Organizational framework conditions	Sufficient	Single item wording	Acceptable
Supervisory quality	Minimally acceptable	Single item wording	Acceptable
Staff support	Acceptable	Single item wording	Acceptable
Peer support	Minimally acceptable	Single item wording	Acceptable
Equal treatment	Acceptable	Single item wording	Acceptable
Monitoring cognition	Sufficient	Single item wording	Acceptable
Control cognition	Not acceptable	Single item wording	Acceptable
Monitoring motivation	Acceptable	Single item wording	Acceptable
Control motivation	Minimally acceptable	Single item wording	Acceptable
Monitoring emotion	Acceptable	Construct definition	Acceptable
Control emotion	Minimally acceptable	Single item wording	Acceptable
Monitoring context	Acceptable	Construct definition	Acceptable
Control context	Minimally acceptable	Single item wording	Acceptable

^a^A reliability value of 0.70 is considered an acceptable lower bound, and values between 0.65 and 0.70 can be considered minimally acceptable. Reliability and information reproduction values cannot be calculated for general items

^b^In cases of limited information reproduction, the recommendation is to refer to the narrower formulation of the single item rather than to the broader formulation of the construct definition for study design and interpretation of the results

## Discussion

In this study, we analyzed the psychometric properties of single items measuring different aspects of undergraduate health sciences students’ self-regulated learning (SRL) in the workplace. First, we assessed the psychometric properties of 29 single items selected from full WLI scales [29], of which 27 items showed sufficient reliability; however, the results regarding validity were heterogeneous. Second, we assessed the psychometric properties of four generally formulated single items [28], which showed acceptable validity, although their reliability could not be assessed. Consequently, this study provides evidence to inform decision-making regarding whether to use single-item measures rather than full scales when investigating the various aspects of workplace learning.

### Single items’ psychometric properties

Reliability was acceptable for most of the 29 single items selected from the WLI. The broad range of reliability results is in line with Gogol et al. [29], who found low-reliability values for different types of academic anxiety and low- to acceptable-reliability values for different types of self-concept. If higher reliability is desired, single items can be used as daily measures aggregated to weekly measures in diary studies.

Regarding the items’ validity in terms of information reproduction, 27 of the 29 selected single items showed limited validity. This result aligns with Gogol et al. [29], who also found low correlations of their anxiety single items with the corresponding full scales (but acceptable correlations of their self-concept single items with the corresponding full scales). The possible reasons for low information reproduction are manifold [52]. For example, the constructs might be too complex [27, 52], and the items may not be representative [28, 29]. That could be the case for the single-item of the construct ‘reviewing’, whose information reproduction was particularly low. Additionally, the response format might not have enough categories and might, therefore, lack sufficient sensitivity [27]. We recommend cautious interpretation of such items, particularly if the items used do not represent the construct, as defined above. For example, although planning, as defined in Table 1, includes both anticipation and planning, the corresponding single item only addresses anticipation, necessitating a narrow interpretation using the single item’s wording.

The 29 single items selected from the WLI showed acceptable validity in terms of similar relationships with external criteria of the nomological network compared to the full scales. Similarly, the absolute differences between the correlations obtained for the full scales and those for the single items were small. The range of mean absolute differences is similar to Gogol et al.’s [29].

For the four newly developed single items, we analyzed how well they correlated with their corresponding WLI full scales and with the external criteria within the nomological network. In summary, the correlations were as expected. For example, the correlations of ‘cognition general’ or ‘motivation general’ with their respective full scales were significant while the correlation with external criteria were not always. This is plausible as the respective full scales were developed for the workplace setting while the scales that measured external criteria were developed for the classroom setting. In contrast, the correlations of ‘context general’ with both respective full scales and external criteria were significant as both were developed for the workplace setting. This study’s results should be used to interpret future studies’ results derived from the newly developed single-item measures. For example, the generally formulated single item “I am motivated today” showed a high correlation with situational interest and the mastery goal approach but a low correlation with the expectancy of success. Hence, the item represents the value rather than the expectancy component of motivation.

For constructs where the single items’ psychometric properties are insufficient, we recommend the use of full scales, such as for ‘reviewing’ and ‘control cognition’ which have low reliability or for further constructs if it is important to include multiple facets of the construct. However, researchers often need to balance the number of constructs measured with ethical standards to avoid overburdening participants, as well as to obtain complete and valid data. Several scenarios might justify the use of single items with limited information reproduction: (1) when the single item represents a control or moderator variable [30], (2) when a narrower definition of the construct is justified and the item represents the study’s aspect of interest [27], or (3) when the measure’s desired precision is low [28, 30].

### Strengths, limitations, and implications

Our study’s strength is its rigorous methodology to test the psychometric properties of 33 single items [28, 29]. Furthermore, our data represent students’ heterogeneity in terms of cognition, motivation, and emotions, as we collected the data from an almost full cohort of students at one institution. The data also represent heterogeneous learning environments, as the students were in very different workplace settings.

A limitation is that our respondents were from two institutions only. This was necessary because we aimed to collect high-quality data using rigorous implementation management, such as ensuring support from all stakeholders and adequate time for the respondents to complete the questionnaires at the workplace. Furthermore, although preliminary analysis showed measurement invariance regarding gender, this result is limited due to the limited number of male participants. Future studies need sufficient participants to test for measurement invariance. Another limitation is the self-report aspect of our measurement instrument, as the reported information can sometimes differ from actual lived experiences [53, 54]. This study was an important first step in assessing the psychometric properties of single items for measuring different aspects of health sciences undergraduates’ SRL at the workplace. Further research should validate the items using alternative measures for comparison. Furthermore, items with different wording should be tested to improve the quality of the single items [28].

The scientific implication of our study lies in its provision of evidence to inform decision-making regarding whether to use scales versus single-item measures to investigate undergraduates’ workplace learning in health sciences education. This study took a very differentiated view of SRL by providing items on its various aspects. Such a differentiated view with corresponding single items enables future researchers to investigate SRL sub-processes. However, the use of single items also makes it possible to consider several aspects of SRL simultaneously and thus take a more holistic view of SRL. This allows researchers to be more economical in their data collection and to include more constructs. It also supports multivariate longitudinal and diary studies of workplace learning.

The research’s practical implication is its contribution to building scientific evidence that can serve as a foundation for developing interventions to enhance workplace learning. The single items can be used for screenings to further probe cognition, motivation, emotions, and contexts in workplace learning within a particular cohort toward the evaluation of different workplace learning curricula. The items can also be used for learning analytics.

## Conclusion

The present study has enhanced knowledge of the psychometric properties of single items measuring different aspects of undergraduates’ self-regulated learning (SRL) at the workplace. Most single-items showed acceptable reliability but the results regarding validity were mixed. While the single-items reproduced the relationships with external criteria in the nomological network that the full scales obtain, most single-items insufficiently reproduced the information that the full scales obtained. The results provide evidence for health sciences education researchers to decide between using full scales and single items. The present study supports further investigation of health sciences undergraduates’ SRL at the workplace.

### Electronic supplementary material

Below is the link to the electronic supplementary material.

Supplementary Material 1

## Data Availability

The datasets used and/or analyzed during the current study are available from the corresponding author upon reasonable request.

## Abbreviations

WLI: Workplace learning inventory in health sciences education
SRL: Self-regulated learning
