# Supplementary material for: Undergraduates’ workplace learning in health sciences education: psychometric properties of single-item measures
Source: BMC Med Educ. 2024 Aug 10;24:861. doi: 10.1186/s12909-024-05848-7 (PMC11316308; doi:10.1186/s12909-024-05848-7)
Supplement: Supplementary file 1 — Supplementary Material 1 [file 12909_2024_5848_MOESM1_ESM.docx]

**Supplement material**

**Table 1**

Correlations, means and standard deviations

| Scale | 1. | 2. | 3. | 4. | 5. | 6. | 7. | 8. | 9. | 10. | 11. | 12. | 13. | 14. | 15. | 16. | 17. | 18. | 19. | 20. | 21. | 22. | 23. | 24. | 25. | 26. | 27. | 28. |  |
| --- | --- | --- | --- | --- | --- | --- | --- | --- | --- | --- | --- | --- | --- | --- | --- | --- | --- | --- | --- | --- | --- | --- | --- | --- | --- | --- | --- | --- | --- |
| 1. Preparation |  |  |  |  |  |  |  |  |  |  |  |  |  |  |  |  |  |  |  |  |  |  |  |  |  |  |  |  |  |
| 2. Attention | 0.34 |  |  |  |  |  |  |  |  |  |  |  |  |  |  |  |  |  |  |  |  |  |  |  |  |  |  |  |  |
| 3. Rehearsal | 0.51 | 0.58 |  |  |  |  |  |  |  |  |  |  |  |  |  |  |  |  |  |  |  |  |  |  |  |  |  |  |  |
| 4. Elaboration | 0.44 | 0.66 | 0.59 |  |  |  |  |  |  |  |  |  |  |  |  |  |  |  |  |  |  |  |  |  |  |  |  |  |  |
| 5. Clarification | 0.41 | 0.66 | 0.62 | 0.61 |  |  |  |  |  |  |  |  |  |  |  |  |  |  |  |  |  |  |  |  |  |  |  |  |  |
| 6. Consolidation | 0.58 | 0.37 | 0.57 | 0.41 | 0.45 |  |  |  |  |  |  |  |  |  |  |  |  |  |  |  |  |  |  |  |  |  |  |  |  |
| 7. Planning | 0.65 | 0.34 | 0.53 | 0.53 | 0.35 | 0.44 |  |  |  |  |  |  |  |  |  |  |  |  |  |  |  |  |  |  |  |  |  |  |  |
| 8. Reviewing | 0.51 | 0.50 | 0.6 | 0.67 | 0.52 | 0.52 | 0.54 |  |  |  |  |  |  |  |  |  |  |  |  |  |  |  |  |  |  |  |  |  |  |
| 9. Reflection | 0.34 | 0.40 | 0.47 | 0.53 | 0.37 | 0.56 | 0.47 | 0.56 |  |  |  |  |  |  |  |  |  |  |  |  |  |  |  |  |  |  |  |  |  |
| 10. Expectancy of success | 0.15 | 0.23 | 0.26 | 0.23 | 0.42 | 0.12 | 0.02 | 0.20 | 0.08 |  |  |  |  |  |  |  |  |  |  |  |  |  |  |  |  |  |  |  |  |
| 11. Situational interest | 0.27 | 0.47 | 0.47 | 0.49 | 0.43 | 0.29 | 0.29 | 0.33 | 0.31 | 0.28 |  |  |  |  |  |  |  |  |  |  |  |  |  |  |  |  |  |  |  |
| 12. Mastery goal approach | 0.38 | 0.55 | 0.52 | 0.64 | 0.52 | 0.34 | 0.36 | 0.43 | 0.40 | 0.22 | 0.72 |  |  |  |  |  |  |  |  |  |  |  |  |  |  |  |  |  |  |
| 13. Performance goal approach | 0.27 | 0.25 | 0.38 | 0.31 | 0.16 | 0.33 | 0.26 | 0.35 | 0.34 | -0.10 | 0.27 | 0.36 |  |  |  |  |  |  |  |  |  |  |  |  |  |  |  |  |  |
| 14. Effort | 0.49 | 0.50 | 0.53 | 0.56 | 0.44 | 0.44 | 0.34 | 0.45 | 0.42 | 0.12 | 0.48 | 0.67 | 0.39 |  |  |  |  |  |  |  |  |  |  |  |  |  |  |  |  |
| 15. Attention control | -0.06 | -0.25 | -0.17 | -0.35 | -0.15 | -0.03 | -0.06 | -0.07 | -0.08 | -0.10 | -0.44 | -0.46 | -0.04 | -0.36 |  |  |  |  |  |  |  |  |  |  |  |  |  |  |  |
| 16. Proactive attitude | 0.21 | 0.54 | 0.41 | 0.49 | 0.50 | 0.22 | 0.20 | 0.34 | 0.28 | 0.25 | 0.58 | 0.63 | 0.30 | 0.51 | -0.24 |  |  |  |  |  |  |  |  |  |  |  |  |  |  |
| 17. Negative emotions | 0.09 | -0.17 | -0.11 | -0.17 | -0.20 | 0.09 | 0.06 | 0.00 | 0.18 | -0.26 | -0.37 | -0.23 | 0.21 | -0.05 | 0.30 | -0.29 |  |  |  |  |  |  |  |  |  |  |  |  |  |
| 18. Positive emotions | 0.26 | 0.42 | 0.39 | 0.51 | 0.39 | 0.24 | 0.26 | 0.42 | 0.37 | 0.30 | 0.62 | 0.51 | 0.14 | 0.37 | -0.35 | 0.43 | -0.36 |  |  |  |  |  |  |  |  |  |  |  |  |
| 19. Organizational framework conditions | 0.16 | 0.27 | 0.30 | 0.36 | 0.26 | 0.18 | 0.18 | 0.2 | 0.13 | 0.24 | 0.55 | 0.44 | 0.06 | 0.26 | -0.22 | 0.34 | -0.44 | 0.40 |  |  |  |  |  |  |  |  |  |  |  |
| 20. Supervisory quality | 0.30 | 0.33 | 0.40 | 0.41 | 0.34 | 0.27 | 0.24 | 0.34 | 0.21 | 0.25 | 0.55 | 0.45 | 0.22 | 0.32 | -0.14 | 0.39 | -0.35 | 0.46 | 0.75 |  |  |  |  |  |  |  |  |  |  |
| 21. Staff support | 0.16 | 0.31 | 0.27 | 0.34 | 0.30 | 0.09 | 0.15 | 0.25 | 0.06 | 0.23 | 0.41 | 0.33 | -0.02 | 0.23 | -0.15 | 0.34 | -0.48 | 0.37 | 0.73 | 0.73 |  |  |  |  |  |  |  |  |  |
| 22. Peer support | 0.13 | 0.25 | 0.12 | 0.20 | 0.26 | 0.00 | 0.01 | 0.09 | -0.05 | 0.22 | 0.21 | 0.16 | -0.06 | 0.13 | -0.04 | 0.24 | -0.29 | 0.17 | 0.47 | 0.47 | 0.54 |  |  |  |  |  |  |  |  |
| 23. Equal treatment | 0.05 | 0.21 | 0.12 | 0.14 | 0.13 | -0.01 | 0.06 | 0.02 | 0.04 | 0.17 | 0.18 | 0.23 | 0.05 | 0.22 | -0.08 | 0.23 | -0.13 | 0.07 | 0.31 | 0.25 | 0.27 | 0.35 |  |  |  |  |  |  |  |
| 24. Motivation Monitoring | 0.20 | 0.25 | 0.28 | 0.36 | 0.32 | 0.24 | 0.29 | 0.39 | 0.39 | 0.19 | 0.27 | 0.35 | 0.28 | 0.25 | -0.10 | 0.24 | 0.17 | 0.34 | 0.09 | 0.10 | -0.06 | -0.03 | -0.12 |  |  |  |  |  |  |
| 25. Motivation Control | 0.36 | 0.29 | 0.41 | 0.42 | 0.33 | 0.34 | 0.37 | 0.41 | 0.27 | 0.19 | 0.35 | 0.37 | 0.24 | 0.36 | -0.35 | 0.26 | -0.1 | 0.52 | 0.23 | 0.31 | 0.18 | 0.09 | -0.08 | 0.47 |  |  |  |  |  |
| 26. Emotion Monitoring | 0.20 | 0.13 | 0.25 | 0.28 | 0.23 | 0.23 | 0.28 | 0.32 | 0.42 | 0.14 | 0.19 | 0.26 | 0.27 | 0.22 | -0.06 | 0.19 | 0.3 | 0.25 | 0.01 | 0.04 | -0.13 | -0.07 | -0.09 | 0.81 | 0.40 |  |  |  |  |
| 27. Emotion Control | 0.19 | 0.34 | 0.31 | 0.41 | 0.38 | 0.13 | 0.20 | 0.26 | 0.19 | 0.20 | 0.32 | 0.36 | 0.09 | 0.35 | -0.42 | 0.36 | -0.25 | 0.45 | 0.16 | 0.16 | 0.12 | 0.12 | -0.06 | 0.35 | 0.73 | 0.35 |  |  |  |
| 28. Context Monitoring | 0.25 | 0.21 | 0.26 | 0.30 | 0.26 | 0.18 | 0.34 | 0.31 | 0.36 | 0.14 | 0.20 | 0.27 | 0.23 | 0.21 | -0.13 | 0.14 | 0.23 | 0.22 | 0.02 | 0.06 | -0.05 | -0.04 | -0.10 | 0.72 | 0.38 | 0.77 | 0.35 |  |  |
| 29. Context Control | 0.25 | 0.29 | 0.39 | 0.38 | 0.30 | 0.34 | 0.32 | 0.39 | 0.40 | 0.18 | 0.31 | 0.36 | 0.21 | 0.32 | -0.22 | 0.28 | 0.02 | 0.39 | 0.07 | 0.18 | 0.04 | -0.03 | -0.11 | 0.46 | 0.59 | 0.50 | 0.63 | 0.55 |  |
| M | 3.39 | 4.31 | 3.64 | 3.78 | 3.89 | 3.01 | 3.56 | 3.22 | 3.39 | 3.73 | 3.97 | 4.18 | 3.36 | 3.92 | 2.47 | 3.73 | 2.07 | 3.31 | 3.74 | 3.97 | 4.11 | 4.45 | 3.52 | 3.07 | 3.04 | 2.73 | 3.17 | 3.11 | 3.11 |
| SD | 1.03 | 0.77 | 0.75 | 0.80 | 0.79 | 1.03 | 0.86 | 0.58 | 1.00 | 0.86 | 0.99 | 0.86 | 0.88 | 0.91 | 0.98 | 0.56 | 0.85 | 0.83 | 0.97 | 0.75 | 0.82 | 0.77 | 0.31 | 1.01 | 0.90 | 1.08 | 0.99 | 1.07 | 2.95 |
